# Characteristics of the Residential Environment and Their Association With Depression in Hong Kong

**DOI:** 10.1001/jamanetworkopen.2021.30777

**Published:** 2021-10-29

**Authors:** Chinmoy Sarkar, Ka Yan Lai, Sarika Kumari, Gabriel M. Leung, Chris Webster, Michael Y. Ni

**Affiliations:** 1 Healthy High Density Cities Lab, HKUrbanLab, The University of Hong Kong, Pokfulam, Hong Kong Special Administrative Region, China; 2 School of Public Health, The University of Hong Kong, Pokfulam, Hong Kong Special Administrative Region, China; 3 The State Key Laboratory of Brain and Cognitive Sciences, The University of Hong Kong, Hong Kong Special Administrative Region, China

## Abstract

**Importance:**

With rapid urbanization, understanding the role of residential environments in the development of depression has gained importance. However, the potential associations of residential space and density with depression have been understudied.

**Objective:**

To investigate longitudinal associations of residential livable space and density with depression.

**Design, Setting, and Participants:**

This longitudinal cohort study used data from 2 waves of the FAMILY Cohort, a population-based cohort in the Hong Kong Special Administrative Region, China, recruited between February 28, 2009, and March 28, 2011, at baseline and followed up between August 3, 2011, and June 19, 2013, at wave 2. Data were analyzed from September 1, 2020, through August 10, 2021. A total of 16 968 participants aged 16 years or older underwent assessment using the Patient Health Questionnaire–9 (PHQ-9) and had complete data across all variables.

**Exposures:**

Residential density was objectively assessed at 3 scales (within apartment, building block, and at neighborhood level) at baseline and follow-up.

**Main Outcomes and Measures:**

Depressive symptoms were defined in terms of a PHQ-9 threshold greater than or equal to 5 and probable major depression with a cutoff value greater than or equal to 10. A continuous PHQ-9 score was used as a secondary outcome. Multilevel logistic regression models were used to examine associations of the residential density with probable major depression and depressive symptoms adjusted for time-varying sociodemographic and lifestyle factors, comorbidities, and other environment variables.

**Results:**

Of the 16 968 participants with complete data, the mean (SD) age was 45.5 (16.7) years, and 9328 participants (55.0%) were women at baseline. The mean follow-up was 2.2 years (range, 1.3-3.6 years). At the household level, after full adjustments, each IQR increment in residential livable area was associated with lower odds of probable major depression (adjusted odds ratio [aOR], 0.84; 95% CI, 0.71-0.98; *P* = .03) and depressive symptoms (aOR, 0.93; 95% CI, 0.86-1.00; *P* = .04). At the building-block level, each IQR increment in block density was associated with higher odds of depressive symptoms (aOR, 1.11; 95% CI, 1.01-1.22; *P* = .03), but only in single-housing environment models. The results remained consistent in models with continuous PHQ-9 evaluation.

**Conclusions and Relevance:**

This study’s findings suggest that residential livable space and block density may play a role in the development of depression. Integrated mental health and housing policies promoting mental capital in cities, such as health-optimized density at the household and building-block levels, might serve as a population approach to reduce the burden of depression.

## Key Points

**Question:**

Are residential environments, specifically livable space and residential density at a building-block and neighborhood level, associated with depression?

**Findings:**

In this cohort study involving 16 968 participants followed up over 2 waves, each IQR increment in livable area was associated with 16% lower odds of probable major depression and 7% lower odds of depressive symptoms. Each IQR increment in building-block density was associated with 11% higher odds of depressive symptoms, only in single-housing environment models.

**Meaning:**

These findings suggest that policies to provide more residential livable space and lower residential density at the building-block scale may be associated with less depression.

## Teaser

This cohort study examines the rate of depression associated with residential livable space in Hong Kong.

## Introduction

Depression is a leading cause of mental health–related disease burden, affecting 4.4% of the global population (322 million people in 2015) and accounting for 54 million years lived with disability, which accounts for 7.5% of all years lived with disability.^1^ The age-adjusted rate of disability-adjusted life-years was estimated at 598 per 100 000 in 2016.^2^ The economic costs attributable to poor mental health were estimated at $2.5 trillion per annum in 2010, a figure projected to increase to $6 trillion by 2030.^3^ Prevention of depressive sequelae may therefore represent one of the beneficial goals to comprehensively improve population health.^4^ With more than half of the global population (approximately 4.2 billion people) currently residing in cities, the role of physical and social environmental determinants of depression have received growing attention.^5,6,7^ Among urban built environment attributes, housing arguably has the strongest potential to affect mental health and well-being over the life-course owing to the considerable time spent at home. However, the role of housing environments—in particular, living density—in depression has been understudied.

At a household level, a UK study found self-reported overcrowding to be associated with poorer mental well-being.^8^ Cross-sectional studies in Asia have reported similar findings.^9,10^ At a mesoscale of residential building, block density has also been used as a marker of crowding. An ecological study in Chicago reported the number of units per block to be the second most important housing feature (following the number of persons per room) associated with social aberrations.^11^ At the macroscale, residential density, defined by the number of dwelling units within a residential catchment (neighborhood), is a well-established measure, often acting as a proxy for exposure to health and welfare-enhancing urban attributes, such as availability of community services, walkability, choice of destinations, social interactions, and livability.^12,13^ A study conducted in the US reported higher Census tract–level residential density to be associated with fewer depressive symptoms.^14^

Nonetheless, studies linking objectively measured residential crowding and density with depression are few and inconclusive owing to a preponderance of small-sample, cross-sectional studies conducted in low-density, homogeneous settings that are underpowered. We aimed to examine the longitudinal association between residential density and depression at 3 spatial scales (within the apartment, building block, and the neighborhood) using the FAMILY Cohort.

## Methods

### Study Design and Participants

We conducted a longitudinal cohort study using data from the baseline and follow-up (wave 2) of the FAMILY Cohort, a population-based cohort study of physical, mental, and social health in Hong Kong. The cohort used family members living in the same household as the fundamental unit of sampling and recruited population-proportionate participants through stratified random sampling across the 18 districts of Hong Kong. The baseline wave comprised 46 001 participants residing in 20 279 households recruited from 2009 to 2011, with 69.2% of the participants followed up in wave 2 during the period from 2011 to 2013. The cohort profile has been described elsewhere.^15^

In the present study, after excluding participants with missing data on outcomes and residential environment exposures, the target sample comprised 39 276 participants aged 16 years or older at baseline; of these, 13 637 participants were lost to follow-up. After further excluding missing data across covariates at baseline (n = 4602) and wave 2 (n = 4069), 16 968 participants were available for complete case analyses (Figure). They were recruited between February 28, 2009, and March 28, 2011, at baseline and followed up between August 3, 2011, and June 19, 2013, at wave 2. The study was approved by the institutional review board of the University of Hong Kong/Hospital Authority Hong Kong-West Cluster. Written informed consent was obtained from all participants, and participants received financial compensation. The study is reported as per the Strengthening the Reporting of Observational Studies in Epidemiology (STROBE) reporting guideline.

**Figure.**
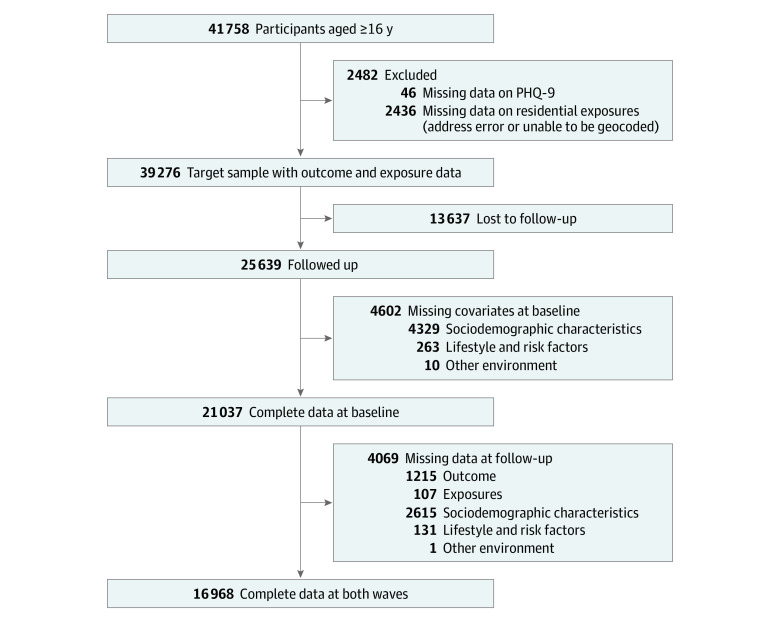
Flowchart Showing the Selection of Participants PHQ-9 indicates Patient Health Questionnaire–9.

### Procedures

The Patient Health Questionnaire–9 (PHQ-9), a 9-item scale used as a standardized diagnostic tool for depressive episodes in the *Diagnostic and Statistical Manual of Mental Disorders, Fifth Edition* was used for the assessment of mental health.^16^ Each item of this screening instrument was scored between 0 and 3 to produce a continuous score ranging from 0 to 27, with higher scores indicating greater severity of depressive symptoms. The overall internal consistency of the PHQ-9 score within the cohort was 0.82, and an interclass correlation for test-retest reliability over a 2-month period was 0.76.^17^ Our primary outcomes comprised binary indicators of depressive symptoms defined in terms of a PHQ-9 threshold greater than or equal to 5 and probable major depression with a cutoff value greater than or equal to 10. A meta-analysis had established that a PHQ-9 score of 10 had a sensitivity of 85% and specificity of 89% for the diagnosis of major depression.^18^

We geocoded 19 719 cohort participants’ (97.2%) home addresses at baseline and 14 113 (93.1%) wave 2 participants to the building-block and floor levels. Residential exposures for the cohort participants were derived from the Hong Kong Housing Environment Database, a geospatial database developed by us and linked to the participants’ geocoded addresses. Microscale livable space, used as a proxy of crowding, was measured by residential floor area in square meters. At a mesoscale, the number of residential units (apartments) in a building block was taken as a measure of block density. The number of residential units per square kilometer, measured within a street-network catchment radius of 402 m (0.25 miles) and 805 m (0.50 miles), signified neighborhood-level (macroscale) density (eFigure; eMethods in the Supplement). Other residential environmental variables included building age, floor level, public transport density, terrain variability (both measured at neighborhood levels of 402- and 805-m street catchments), and proportion of the working population.

### Statistical Analysis

We excluded all individuals younger than 16 years. Multilevel models are well suited to analyze longitudinal data, with inherent ability to account for underlying correlations in data, for example, correlations between repeated measurements of outcome and exposures and those attributable to contextual factors, such as clustering within households. We fitted multilevel logistic regression models with a robust variance estimator to examine the longitudinal associations of the residential density measured at 402-m street catchment with probable major depression and depressive symptoms using the baseline and follow-up (wave 2) waves of the cohort. A series of 3-level, fixed-effects models were developed with the repeated measurements of depressive sequelae (at baseline and wave 2) nested within individual participants and participants within households, with random intercepts for individual participants and households. Two sets of models were developed. In the single-housing environment models, the density metrics of livable floor area, housing units per building block, and neighborhood residential density were entered into the models separately; in a composite model of multiple housing environments, all 3 metrics were simultaneously entered into the models. The models were sequentially adjusted for covariates and confounders examined at the 2 measurement points. Covariates and confounders were identified from the literature based on assumed associations^19^ and included sociodemographic, lifestyle, comorbidity, and housing environment variables (eMethods in the Supplement). Minimally adjusted (for age and sex), moderately adjusted (for sociodemographic characteristics [including age and sex], lifestyle, and comorbidities), and fully adjusted (for sociodemographic characteristics, lifestyle, comorbidities, and residential environment) models were analyzed.

We conducted several sensitivity tests to examine the robustness of the results. Multiple imputation by chained equations was conducted in Stata, version 16 (StataCorp LLC) to account for missing data.^20^ We imputed missing data attributable to loss to follow-up and missingness across covariates by including all outcomes, exposures, and covariates at baseline and wave 2 in our imputation models for a target sample of 39 276 participants. We reran the multilevel models upon the created 20 imputation sets.^21,22^ We also examined the associations between housing environment variables and PHQ-9 score (as a marker of depressive symptoms). To account for the positive skewness observed in the PHQ-9 scores, multilevel negative binomial regression models with random intercepts for individual participants and households were developed. We further examined associations with the built environment measured at a larger scale of 805 m of residential street catchment. To examine the association of housing environment with depression in wave 2 by depression status at baseline, we conducted multinomial logit regression models. We conducted subgroup-level analyses by age, sex, and employment status to examine potential effect modification between livable area and depressive sequelae and tested for interaction effects. In addition, we repeated the analyses for participants who did not change residential address (nonmovers). All analyses were performed in Stata, version 16.^23^ All significance tests were 2-tailed, and statistical significance was set at *P* < .05

## Results

This analysis comprised 16 968 participants with a mean (SD) age at baseline of 45.5 (16.7) years. The baseline population included 9328 women (55.0%) and 7640 men (45.0%), contributing 36 911.7 person-years of follow-up over a mean of 2.2 years (range, 1.3-3.6 years). Prevalent cases of depressive symptoms were present in 11.0% of the cohort or 1872 participants (in 1.9%, 323 participants for probable major depression) at baseline and 7.6% or 1283 participants (in 1%, 162 participants for probable major depression) at wave 2. The mean (SD) livable area was 43.2 (14.4) m^2^ at baseline and 42.7 (14.5) m^2^ at wave 2; the mean number of housing units per block was 467 (297) at baseline and 468 (296) at wave 2. The mean neighborhood residential density within a 402-m neighborhood catchment was 38 026 (14 570) units/km^2^ at baseline and 38 190 (14 661) units/km^2^ at wave 2. Complete characteristics are presented in Table 1.

**Table 1.** Descriptive Characteristics of 16 968 Participants in the FAMILY Cohort

Characteristic	Participants, No. (%)	
	Baseline	Wave 2
Sociodemographic covariate		
Age, mean (SD), y	45.5 (16.7)	47.7 (16.6)
Sex		
Female	9328 (55.0)	NA
Male	7640 (45.0)	NA
Marital status		
Never married	3965 (23.4)	3731 (22.0)
Married	11 714 (69.0)	11 661 (68.7)
Widowed/divorced/separated	1289 (7.6)	1576 (9.3)
Personal income, HK$^a^		
<5000	6952 (41.0)	6118 (36.1)
5000-9999	3467 (20.4)	3208 (18.9)
10 000-14 999	2660 (15.7)	3035 (17.9)
≥15 000	3889 (22.9)	4607 (27.2)
Educational level		
Primary	3740 (22.0)	3744 (22.1)
Secondary	8229 (48.5)	7816 (46.1)
Tertiary	4999 (29.5)	5408 (31.9)
Employment status		
Employed	9063 (53.4)	9618 (56.7)
Unemployed, homemaker, other	5469 (32.2)	4859 (28.6)
Retiree/unemployed	2436 (14.4)	2491 (14.7)
Lifestyle and comorbidities		
No. of family members		
Living alone	1999 (11.8)	2009 (11.8)
2	5792 (34.1)	5998 (35.3)
3	4167 (24.6)	4229 (24.9)
≥4	5010 (29.5)	4732 (27.9)
Smoking status		
Nonsmoker/past smoker	14 756 (87.0)	14 991 (88.3)
Current smoker	2212 (13.0)	1977 (11.7)
Alcohol consumption		
Never/former	12 757 (75.2)	13 028 (76.8)
1-3/mo	3060 (18.0)	2882 (17.0)
1-3/wk to daily	1151 (6.8)	1058 (6.2)
Religion		
None	11 965 (70.5)	11 721 (69.1)
Christianity/Roman Catholicism	2973 (17.5)	2961 (17.5)
Buddhism/other	2030 (12.0)	2286 (13.5)
Self-reported coronary heart disease		
No	16 591 (97.8)	16 610 (97.9)
Yes	377 (2.2)	358 (2.1)
Self-reported high cholesterol level		
No	15 718 (92.6)	15 914 (93.8)
Yes	1250 (7.4)	1054 (6.2)
Other neighborhood environment		
Residential building age, year built		
1945-1980	3638 (21.4)	3577 (21.1)
1981-1995	7107 (41.9)	7088 (41.8)
Post-1995	6223 (36.7)	6303 (37.1)
Public transport density, median (IQR), U/km^2^		
Within 402 m	37.8 (23.9-56.8)	37.6 (23.9-56.5)
Within 805 m	36.8 (26.7-49.1)	36.7 (26.6-48.9)
Terrain, median (IQR), mean slope in degrees		
Within 402 m	5.5 (2.2-11.7)	5.5 (2.2-11.8)
Within 805 m	7.4 (3.5-12.5)	7.4 (3.5-12.6)
Neighborhood cohesion^b^		
Low	1273 (7.5)	825 (4.9)
Medium	12 827 (75.6)	12 220 (72.0)
High	2868 (16.9)	3923 (23.1)
Proportion of working population in neighborhood, mean (SD)	49.4 (6.3)	49.4 (6.3)
Housing environment variables		
Floor area, mean (SD), m^2^	43.2 (14.4)	42.7 (14.5)
Housing units per building block, mean (SD)	467.2 (296.7)	468.2 (296.4)
Neighborhood residential density, mean (SD), U/km^2^		
Within 402 m	38 025.9 (14 570.1)	38 189.5 (14 660.7)
Within 805 m	29 102.9 (11 325.1)	29 496.9 (11 246.1)
Floor level		
0-5	3156 (18.6)	3122 (18.4)
6-10	3207 (18.9)	3191 (18.8)
11-20	5100 (30.1)	5089 (30.0)
≥21	5505 (32.4)	5566 (32.8)

Abbreviation: NA, not applicable.

^a^ HK $1.0 to US $0.13.

^b^ Neighborhood cohesion score is based on a 5-item questionnaire on a neighbor's willingness to help, being close-knit, trustworthiness, ablility to get along, and sharing similar values. The composite score was recoded as a 3-factor variable (low, medium, and high).

Longitudinal models examining the association between single-housing environment exposure and depressive sequelae are presented in Table 2. Each IQR increment in livable floor area was associated with lower odds of probable major depression (adjusted odds ratio [aOR], 0.82; 95% CI, 0.70-0.96; *P* = .01) and depressive symptoms (aOR, 0.91; 95% CI, 0.85-0.98; *P* = .009) after full adjustments. However, higher building-block density was found to be independently associated with higher odds of depressive symptoms (aOR, 1.11; 95% CI, 1.01-1.22; *P* = .03) (ie, every IQR increment of 490 units at the mesoscale of building block was associated with 11% higher odds of depressive symptoms). Table 3 (eTable 2 in the Supplement provides the full model) presents the composite multilevel models of longitudinal association between multiple housing environment exposures (with the 3 measures of residential density; within-apartment floor area, building-block density, and neighborhood residential density introduced simultaneously in the same model) and depressive sequelae. After full adjustments, every IQR increase in livable floor area was associated with lower odds of probable major depression (aOR, 0.84; 95% CI, 0.71-0.98; *P* = .03) and depressive symptoms (aOR, 0.93; 95% CI, 0.86-1.00; *P* = .04). Both building-block density and neighborhood residential density within 402 m remained nonsignificant in our fully adjusted models. The household-level accounted for 13.7% of the variances for probable major depression and 14.7% of the variances for depressive symptoms.

**Table 2.** Multilevel Models of Longitudinal Association Between Single-Housing Environment Exposure and Depressive Sequelae Among 16 968 FAMILY Cohort Participants

Variable	Floor area, m^2^ (per IQR)		Housing units per building block (per IQR)		Neighborhood residential density (402 m, per IQR), U/km^2^	
	OR (95% CI)	*P* value	OR (95% CI)	*P* value	OR (95% CI)	*P* value
Probable major depression						
Model 1^a^	0.76 (0.65-0.88)	<.001	1.29 (1.09-1.54)	.004	0.99 (0.87-1.12)	.87
Model 2^b^	0.83 (0.72-0.96)	.01	1.18 (0.99-1.41)	.07	0.98 (0.86-1.12)	.78
Model 3^c^	0.82 (0.70-0.96)	.01	1.20 (0.98-1.47)	.09	0.98 (0.85-1.12)	.73
Depressive symptoms						
Model 1^a^	0.89 (0.83-0.95)	<.001	1.14 (1.05-1.25)	.002	1.03 (0.96-1.09)	.41
Model 2^b^	0.92 (0.87-0.99)	.02	1.09 (1.00-1.19)	.05	1.02 (0.96-1.09)	.47
Model 3^c^	0.91 (0.85-0.98)	.009	1.11 (1.01-1.22)	.03	1.02 (0.95-1.08)	.64

Abbreviation: OR, odds ratio.

^a^ Model 1 adjusted for age and sex.

^b^ In addition to age and sex, model 2 was adjusted for other sociodemographic characteristics (marital status, employment status, educational level, and income), lifestyle (smoking status, alcohol intake frequency, number of family members, and current religion), and comorbidities (cardiac heart disease, high cholesterol level).

^c^ Fully adjusted model 3 comprised the factors in models 1 and 2 plus the residential environment (residential building age, floor level, density of public transport, terrain, neighborhood cohesion, and proportion of working population in the neighborhood). The residential environment (neighborhood residential density, density of public transport, and terrain) was measured within 402 m of the street catchment of participants’ geocoded residences.

**Table 3.** Multilevel Composite Models of Longitudinal Association Between Multiple Housing Environment Exposures and Depressive Sequelae Among 16 968 FAMILY Cohort Participants

Composite housing environment	Probable major depression		Depressive symptoms	
	OR (95% CI)	*P* value	OR (95% CI)	*P* value
Model 1^a^				
Floor area (per IQR), m^2^	0.79 (0.67-0.92)	.003	0.91 (0.85-0.97)	.008
Housing units per building block (per IQR)	1.18 (0.98-1.42)	.08	1.09 (1.00-1.20)	.06
Neighborhood residential density (402 m, per IQR), U/km^2^	0.94 (0.82-1.07)	.34	1.00 (0.94-1.07)	.97
Model 2^b^				
Floor area (per IQR), m^2^	0.85 (0.74-0.99)	.04	0.94 (0.87-1.01)	.07
Housing units per building block (per IQR)	1.12 (0.92-1.35)	.25	1.06 (0.96-1.16)	.24
Neighborhood residential density (402 m, per IQR), U/km^2^	0.95 (0.83-1.09)	.46	1.01 (0.94-1.07)	.82
Model 3^c^				
Floor area (per IQR), m^2^	0.84 (0.71-0.98)	.03	0.93 (0.86-1.00)	.04
Housing units per building block (per IQR)	1.13 (0.91-1.40)	.28	1.07 (0.97-1.19)	.17
Neighborhood residential density (402 m, per IQR), U/km^2^	0.94 (0.82-1.09)	.45	1.00 (0.94-1.07)	.98

Abbreviation: OR, odds ratio.

^a^ Model 1 adjusted for age and sex.

^b^ In addition to age and sex, model 2 was adjusted for other sociodemographic characteristics (marital status, employment status, educational level, and income), lifestyle (smoking status, alcohol intake frequency, number of family members, and current religion), and comorbidities (cardiac heart disease and high cholesterol level).

^c^ Fully adjusted model 3 comprised the factors in models 1 and 2 plus the residential environment (residential building age, floor level, density of public transport, terrain, neighborhood cohesion, and proportion of working population in the neighborhood). The residential environment (neighborhood residential density, density of public transport, and terrain) was measured within 402 m of the street catchment of participants’ geocoded residences.

### Sensitivity Analyses

Repeating analyses with imputed data to account for missingness across covariates and due to loss to follow-up produced consistent results as in our primary analyses (eTable 3 in the Supplement). Our study did not find a systematic difference between the analytic sample and subsample lost (Cohen *d* and *w* values being low for most variables) (eTable 1 in the Supplement). In our composite multilevel negative binomial regression models using continuous PHQ-9 scores with multiple housing environment exposures, each IQR increment in livable floor area was associated with a lower PHQ-9 score subsequent to full adjustments (incidence rate ratio, 0.97; 95% CI, 0.94-1.00; *P* = .050), while every IQR increment in building-block density was associated with a higher PHQ-9 score (incidence rate ratio, 1.06; 95% CI, 1.01-1.10; *P* = .01) (eTable 4 in the Supplement). Repeating all of our analyses with neighborhood residential density and other environmental factors measured at the scale of 805 m residential street catchment produced consistent significant results for livable floor area (eTable 5 in the Supplement). The results of multinomial logit models examining the relative risk of depressive symptoms at wave 2 by depression status at baseline showed that higher livable floor area remained associated with a lower risk of incident depressive symptoms (relative risk, 0.89; 95% CI, 0.81-0.99; *P* = .03). However, none of the housing environment exposures was significant among participants with depressive symptoms at both baseline and wave 2 (eTable 6 in the Supplement). Modifications (by sex, age, and employment status) of associations between livable floor area and depressive sequelae at baseline are presented in eTable 7 in the Supplement. There was no evidence of a significant interaction by sex and age groups. The association between livable floor area and depressive symptoms was more pronounced among the employed participants (employed: aOR, 0.87; 95% CI, 0.78-0.96; *P* = .01; unemployed, homemaker, or others: aOR, 1.02; 95% CI, 0.90-1.16; *P* = .72; retiree/unemployed: aOR, 0.97; 95% CI, 0.83-1.14; *P* = .73; *P* = .02 for interaction). Among the cohort subgroup who did not change residential address over the follow-up period (n = 16 407), each IQR increment in livable floor area was associated with lower odds of probable major depression (aOR, 0.82; 95% CI, 0.70-0.98; *P* = .03) (eTable 8 in the Supplement) and depressive symptoms (aOR, 0.91; 95% CI, 0.84-0.98; *P* = .02).

## Discussion

In this large, longitudinal cohort study conducted in one of the highest-density cities in the world, we found that livable floor area was associated with lower odds for depressive symptoms and probable major depression; building-block density was associated with higher odds of depressive symptoms. Our findings remained robust in models with neighborhood residential density and other neighborhood environment variables assessed at the scale of 805 m and other sensitivity tests.

At the microscale of indoor housing environment, larger livable floor area, used as a marker of the amount of private space, was associated with better mental health, with each IQR increment in floor area (17 m^2^) associated with a 16% lower odds of probable major depression and 7% lower odds of depressive symptoms, consistent with evidence of human and animal studies.^11,24,25,26^ This association remained consistent in our negative binomial regression models with continuous PHQ-9 scores and in models with residential environment measured at a larger scale of 805 m catchment. To our knowledge, this study is the first to estimate this association with private space while controlling for other density metrics that might confound an unadjusted model. This finding is important because the 2 additional density measures we evaluated (at the building-block and neighborhood levels) may have different pathways and work in opposite directions. Each IQR increment in housing units per building block (490 units) at the mesoscale was associated with 11% higher odds of depressive symptoms over the follow-up period only in the single-housing environment models. The results remained consistent in our negative binomial regression models with both single and multiple housing environment exposures.

Laboratory animal experiments have consistently reported associations of crowding and limited livable space with higher risks of depression, deviant behavior, and stress. In one of the earliest animal experiments, Calhoun^24^ showed that rats residing in crowded environments developed social abnormalities, such as unwanted social contact, social withdrawal, aggressive behavior, and higher mortality rate. Monkeys and sows living with reduced space allowances have been shown to have higher levels of hair and plasma cortisol concentrations, indicating chronic stress.^25,26^ Human studies conducted in Chicago showed that the number of people per room and the number of residential units per block appeared to be key determinants of social aberrations, such as higher rates of mortality, admissions to mental hospitals, and juvenile delinquency.^11^ By controlling for objectively measured building and neighborhood density in our models of privately consumed (livable) space, our results demonstrate that the *Calhoun effect* as evident in rodents may also be present in humans.

It is plausible that our associations of livable area and building-block density (microscale and mesoscale) with depression could be mediated by psychosocial stress related to crowding and density. Under the social stress paradigm, chronic psychosocial stress can initiate cognitive and biological processes that elevate the risk of depression.^27,28^ The links between residential overcrowding and density and stress have been previously established.^29,30,31^ Crowded household space and shared space at the building block (architectural scale) may escalate undesirable contacts and stimuli in individuals, leading to poor interpersonal relationships and stress.^11^ If unwanted interactions and the deprivation of privacy are regulated by social norms and rules, the regulation regimen itself may be an additional source of chronic stress and depression.^30^

The protective association of livable floor space with depressive symptoms was more pronounced among participants who were employed, suggesting the importance of personal space given they spend lesser time in their residences compared with those who are unemployed, homemakers, or retired. Our multinomial logit models showed a protective association between floor area and incident depressive symptoms (ie, participants who were free from depressive symptoms at baseline but developed them during the follow-up period) but not among participants who had depressive symptoms both at baseline and wave 2. This null association among the group with persistent depression may be due to low statistical power on account of fewer cases and thus points to the need to conduct further studies in populations with persistent depression.

At a macroscale, neighborhood residential density was not significant in our primary analyses. Models with imputed data reported a protective association, with 11% lower odds of probable major depression per IQR increment. Greater neighborhood residential density has been associated with greater walkability,^12,13^ sense of community, accrued social capital, and higher levels of social interactions, with consequent positive influences on mental health.^32,33^

From a clinical perspective, special attention can be paid to populations exposed to environmental stressors, in particular those residing in buildings of high density and with suboptimal livable space, when screening for people with depression and related mental disorders. With respect to mental health service allocation, our findings may imply that neighborhoods with high-density buildings and limited household space allocation can be a focus, especially when strategizing and designing preventive, tailor-made psychiatric interventions to prevent relapse of depression among people with a history of depression, although more research is needed in this direction.

### Strengths and Limitations

This study has strengths and limitations. Among the strengths, we were able to leverage high-quality data collected over 2 waves of a large, prospective health cohort in one of the highest-density cities of the world. We used standard diagnostic criteria based on the PHQ-9 scale to define depressive sequelae, with scale reliability tested on the local population. The study is, to our knowledge, the first to systematically use multiscalar, objectively measured metrics of residential crowding and density (livable floor area of dwelling, block-level and neighborhood-level residential density) to establish independent associations with depression. Our individual level of analysis overcomes methodological shortcomings, such as modifiable areal unit problems and ecological fallacies. Some studies had used subjectively defined, self-reported measures of living environment or perceptions of it. Advantages of objectively measured, standardized metrics of density include robustness in the exposure-outcome effect estimates and greater replicability and comparability between studies.^34,35^

The limitations of our study stem from an observational design that precludes establishing causality. Nonetheless, longitudinal analyses over 2 time points enabled examinations of the associations of multiscalar housing environments with depression after rigorous adjustments. We were able to conduct a range of sensitivity tests, which are generally not possible or are underpowered in small-scale cross-sectional studies. We acknowledge that the original cohort was susceptible to selection bias. The FAMILY Cohort enrolled complete households in which all adult members agreed to participate, thus potentially selecting better functioning family units with less depressive symptoms.^36^ Our study lacked systematic data on the family history of depression and thus could not adjust for this factor. The results of the study cannot be generalized; studies in other populations with different density profiles and sociocultural contexts are necessary.

## Conclusions

By leveraging Hong Kong’s highly urbanized context, our findings suggest that enhancing living environments by providing adequate residential livable space and health-optimized allocations of housing units at the building-block and neighborhood scale might be a potential population health approach for lowering the rates of depression. With large populations exposed to residential density, even small effects associated with optimizing livable space and density might lead to substantial population health improvements.^37,38^

## Notes

Supplement. **eMethods.** Environment Exposures, Covariates and Confounders **eTable 1.** Comparison of the Baseline Characteristics of the Analytic and Excluded Sample of FAMILY Cohort Participants **eTable 2.** Full Multilevel Models of Longitudinal Association Between Multiple Housing Environment Exposures and Depressive Sequelae Among FAMILY Cohort Participants Aged 16 Years or Older Across Two Waves (N = 16 968) **eTable 3.** Full Multilevel Models of Longitudinal Association Between Composite Housing Environment Exposures and Depressive Sequelae Using Multiple Imputation to Impute Loss to Follow-up and Missingness Across Key Covariates (N = 39 276) **eTable 4.** Multilevel Negative Binomial Regression Models of Longitudinal Association Between Housing Environment Exposures and PHQ-9 Score Among FAMILY Cohort Participants Aged 16 Years or Older Across Two Waves (N = 16 968) **eTable 5.** Multilevel Models of Longitudinal Association Between Composite Housing Environment Exposures and Depressive Sequelae Among FAMILY Cohort Participants Aged 16 Years or Older Across Two Waves With Residential Environment Measured Within 0.50-Mile (805 m) Street Catchment (N = 16 968) **eTable 6.** Results of the Multinomial Logit Regression Models of Association of Housing Environment Exposures and Depressive Symptoms at wave 2 by Depressive Symptoms at Baseline (N = 16 968) **eTable 7.** Association of Livable Floor Area With Prevalent Probable Major Depression and Depressive Symptoms by Population Sub-groups of Sex, Age and Employment Status at Baseline (N = 16 968) **eTable 8.** Multilevel Association of Housing Environment Exposures With Depressive Sequelae Among FAMILY Cohort Participants Aged 16 Years or Older Who Did Not Change Residential Address Over the Study Period (N = 16 407) **eFigure.** An Illustration Showing the Attributes of Housing Exposures of Multi-scalar Residential Space and Density in the Developed HKHED Database
