# Supplementary material for: Characteristics of the Residential Environment and Their Association With Depression in Hong Kong
Source: JAMA Netw Open. 2021 Oct 29;4(10):e2130777. doi: 10.1001/jamanetworkopen.2021.30777 (PMC8556618; doi:10.1001/jamanetworkopen.2021.30777)
Supplement: Supplement. — eMethods. Environment Exposures, Covariates and Confounders eTable 1. Comparison of the Baseline Characteristics of the Analytic and Excluded Sample of FAMILY Cohort Participants eTable 2. Full Multilevel Models of Longitudinal Association Between Multiple Housing Environment Exposures and Depressive Sequelae Among FAMILY Cohort Participants Aged 16 Years or Older Across Two Waves (N = 16 968) eTable 3. Full Multilevel Models of Longitudinal Association Between Composite Housing Environment Exposures and Depressive Sequelae Using Multiple Imputation to Impute Loss to Follow-up and Missingness Across Key Covariates (N = 39 276) eTable 4. Multilevel Negative Binomial Regression Models of Longitudinal Association Between Housing Environment Exposures and PHQ-9 Score Among FAMILY Cohort Participants Aged 16 Years or Older Across Two Waves (N = 16 968) eTable 5. Multilevel Models of Longitudinal Association Between Composite Housing Environment Exposures and Depressive Sequelae Among FAMILY Cohort Participants Aged 16 Years or Older Across Two Waves With Residential Environment Measured Within 0.50-Mile (805 m) Street Catchment (N = 16 968) eTable 6. Results of the Multinomial Logit Regression Models of Association of Housing Environment Exposures and Depressive Symptoms at wave 2 by Depressive Symptoms at Baseline (N = 16 968) eTable 7. Association of Livable Floor Area With Prevalent Probable Major Depression and Depressive Symptoms by Population Sub-groups of Sex, Age and Employment Status at Baseline (N = 16 968) eTable 8. Multilevel Association of Housing Environment Exposures With Depressive Sequelae Among FAMILY Cohort Participants Aged 16 Years or Older Who Did Not Change Residential Address Over the Study Period (N = 16 407) eFigure. An Illustration Showing the Attributes of Housing Exposures of Multi-scalar Residential Space and Density in the Developed HKHED Database [file jamanetwopen-e2130777-s001.pdf]

Supplementary Online Content

Sarkar C, Lai KY, Kumari S, Leung GM, Webster C, Ni MY. Characteristics of the residential environment and their association with depression in Hong Kong. *JAMA Netw Open*. 2021;4(10):e2130777. doi:10.1001/jamanetworkopen.2021.30777

eMethods. Environment Exposures, Covariates and Confounders

eTable 1. Comparison of the Baseline Characteristics of the Analytic and Excluded Sample of FAMILY Cohort Participants

eTable 2. Full Multilevel Models of Longitudinal Association Between Multiple Housing Environment Exposures and Depressive Sequelae Among FAMILY Cohort Participants Aged 16 Years or Older Across Two Waves (N = 16 968)

eTable 3. Full Multilevel Models of Longitudinal Association Between Composite Housing Environment Exposures and Depressive Sequelae Using Multiple Imputation to Impute Loss to Follow-up and Missingness Across Key Covariates (N = 39 276)

eTable 4. Multilevel Negative Binomial Regression Models of Longitudinal Association Between Housing Environment Exposures and PHQ-9 Score Among FAMILY Cohort Participants Aged 16 Years or Older Across Two Waves (N = 16 968)

eTable 5. Multilevel Models of Longitudinal Association Between Composite Housing Environment Exposures and Depressive Sequelae Among FAMILY Cohort Participants Aged 16 or Older Across Two Waves With Residential Environment Measured Within 0.50-Mile (805 m) Street Catchment (N = 16 968)

eTable 6. Results of the Multinomial Logit Regression Models of Association of Housing Environment Exposures and Depressive Symptoms at Wave 2 by Depressive Symptoms at Baseline (N = 16 968)

eTable 7. Association of Livable Floor Area With Prevalent Probable Major Depression and Depressive Symptoms by Population Sub-groups of Sex, Age and Employment Status at Baseline (N = 16 968)

eTable 8. Multilevel Association of Housing Environment Exposures With Depressive Sequelae Among FAMILY Cohort Participants Aged 16 Years or Older Who Did Not Change Residential Address Over the Study Period (N = 16 407)

eFigure. An Illustration Showing the Attributes of Housing Exposures of Multi-scalar Residential Space and Density in the Developed HKHED Database

This supplementary material has been provided by the authors to give readers additional information about their work.

© 2021 Sarkar C et al. *JAMA Network Open.*

eMethods. Environment exposures, covariates and confounders.

Housing density exposure - Hong Kong Housing Environment Database (HKHED)

We developed the Hong Kong Housing Environment Database (HKHED), a geospatial database of objectively measured residential density at different scales, namely within apartment at household level, building block, and neighbourhood level). Spatial data for building block footprints, housing estate boundaries, street centerline and city-wide housing address dataset sourced from the Hong Kong Lands Department (spatial database IG1000), as well as detailed information on housing units within individual blocks, sourced from multiple government and private agencies including The Hong Kong Housing Authority (HA), Hong Kong Housing Society (HS), Hong Kong government's GeoInfo Map, Home Affairs Department and Centaline Property Agency Limited (CentaMap). We created 3 multi-scalar metrics of crowding and density:

- residential liveable space, expressed as liveable floor area of the apartment of the cohort participant (at the micro-level of household),
- building block density, measured as the number of residential units in building (apartment) block of the cohort participant (at a meso-level), and
- neighbourhood housing density at a macro scale, expressed as the number of residential (apartment) units within pre-specified street catchments of 402 m (0.25-mile) and 805 m (0.5-mile). These radii correspond to distance covered in a 5-10 minute walk from a residence in high density Hong Kong, and have been employed elsewhere in studies of mental health[1,2]. We used Hong Kong LANDS Department's street centerline data to create street network catchments around geocoded cohort participants' home address and residential density (in units per square kilometer) was measured as the number of residential units divided by the catchment area.

The full dataset employed to measure participants' neighborhood residential density comprised 2.5 and 2.6 million housing units in the years of the end of baseline and wave 2 respectively. Our models measured the associations between one interquartile range increment in the density exposures and depressive sequelae.

© 2021 Sarkar C et al. *JAMA Network Open.*

Other environment

Among the other environment variables, building age was measured from the first occupation date of each building block and expressed as 1945-80 (old), 1981-95, post-1995 (newly built). Floor level was extracted from the participants' address data. The floor level data had to be further corrected for participants residing in floors that start with "4" (i.e. 40-49), end with "4" (i.e. 4, 14, 24, 34...) and floor level 58, mostly in private housing blocks constructed after the mid-1990s.These building blocks skipped these floor levels due to auspicious reasons in Hong Kong context and adjustments were accordingly made after thorough checking. Floor level was coded as a four-factor variable (0-5, 6-10, 11-20 and >20). The density of public transport was measured as the number of public transport (bus, green minibus, mass transit railway (MTR), light rail, trams and ferries) stops and stations per square kilometer within 402 m and 805 m residential catchments. Terrain is a measure of physical environment that among other things, increases the friction of travel. It was measured from a 5-metre resolution digital terrain model as mean slope within 402 m and 805 m residential catchments. Building age, floor level, density of public and terrain were assessed both at baseline and wave 2. The proportion of working population in the neighbourhood, a proxy of neighbourhood-level economic activity, was measured at the level of large street block groups (LSBG) from the 2011 population census. LSBGs are the lower level census aggregates containing a mean population of approximately 4,350 people in Hong Kong. The data was sourced from the Hong Kong Census and Statistics Department on enquiry and subsequently matched with the participants' geocoded locations.

Covariates and confounders.

In our study, all covariates and confounders (except sex) were measured at two time points, derived from the baseline and wave 2 survey questionnaires. These included socio-demographic covariates (age, sex, marital status, educational qualification, employment status and personal income); lifestyle (number of family members, smoking status, alcohol intake frequency, current religion and neighbourhood cohesion score) and comorbidities (doctor-diagnosed coronary heart disease and high cholesterol) and housing environment attributes (building age, floor level, and public transport density and terrain variability). The cohort participants' age (in years) was calculated from the dates of birth and examination dates at baseline and wave 2. Sex was assessed at baseline. Marital status was derived from a questionnaire: 'What is your current marital status?' and coded as never married; married; and

© 2021 Sarkar C et al. *JAMA Network Open.*

widowed/divorced/separated. Educational level was coded as a 3-factor coded as primary; secondary; and tertiary or higher degrees. Employment status was coded as employed; homemaker/student/others; and retiree/unemployed. Income was derived from the question: ‘Including all your income sources and MPF contribution, what is your average monthly personal income? (in Hong Kong dollars; 1HK$=0.13$US)’ and coded as a 4-factor variable as <HK$ 5,000; 5,000-9,999; 10,000-14,999; and ≥15,000. Among the lifestyle level variables, the number of family members was coded as a 4-factor variable as one, two, three, ≥four. Smoking status was expressed as non-smoker/past smoker versus current smoker, while alcohol intake frequency was a 3-factor variable coded as non-drinker/former drinker; occasional to 1-3 per month; and 1-3 per week up to daily). Religion was based on the question: ‘What, if any, is your religion currently?’ and coded as None; Christianity; and Buddhism/others. Neighbourhood cohesion score based on a five-item questionnaire on neighbour's willingness to help, being close-knit, trustworthy, able to get along and sharing similar values was coded as low, medium and high. Self-reported doctor-diagnosed comorbidities (coronary heart disease and high cholesterol) were coded as binary outcomes (Yes versus No).

1. Berke EM, Gottlieb LM, Moudon AV, Larson EB. Protective Association Between Neighborhood Walkability and Depression in Older Men. *Journal of the American Geriatrics Society*. 2007;55(4):526-533. doi:https://doi.org/10.1111/j.1532-5415.2007.01108.x

2. Hipp J. What is the ‘Neighbourhood’ in Neighbourhood Satisfaction? Comparing the Effects of Structural Characteristics Measured at the Micro-neighbourhood and Tract Levels. *Urban Studies*. 2010;47(12):2517-2536. doi: https://doi.org/10.1177/0042098009359950

© 2021 Sarkar C et al. *JAMA Network Open.*

eTable 1. Comparison of the baseline characteristics of the analytic and excluded sample of FAMILY Cohort participants.

**Participant characteristics**	**Analytic sample (N=16,968)**	**Excluded sample due to missingness on covariates (N=8,671)**	**Effect size[abc]**	**Excluded sample due to loss to follow-up (N=13,637)**	**Effect size[abd]**
Socio-demographic covariates:					
Age in years (Mean, SD)	45.5 (16.7)	52.0 (17.9)	0.38	45.6 (18.2)	0.007
Sex N (%): Female	9,328 (55.0)	4,955 (57.1)	0.02	7,165 (52.5)	0.02
Male	7,640 (45.0)	3,716 (42.9)		6,472 (47.5)	
Marital status N (%): Never married	3,965 (23.4)	1,643 (19.0)	0.10	4,117 (30.2)	0.06
Married	11,714 (69.0)	5,823 (67.5)		8,281 (60.8)	
Widowed/Divorced/separated	1,289 (7.6)	1,167 (13.5)		1,217 (8.9)	
Personal income (HK$[e]) N (%): <5 000	6,952 (41.0)	4,231 (62.3)	0.20	5,629 (44.8)	0.06
5 000-9 999	3,467 (20.4)	1,142 (16.8)		2,427 (19.3)	
10 000-14 999	2,660 (15.7)	679 (10.0)		2,179 (17.3)	
≥15 000	3,889 (22.9)	738 (10.9)		2,332 (18.6)	
Educational level N (%): Primary	3,740 (22.0)	3,078 (36.0)	0.16	3,349 (24.7)	0.04
Secondary	8,229 (48.5)	3,877 (45.3)		6,639 (49.0)	
Tertiary	4,999 (29.5)	1,603 (18.7)		3,563 (26.3)	
Employment status N (%): Employed	9,063 (53.4)	3,025 (49.6)	0.06	6,830 (55.6)	0.02
Unemployed, home maker, others	5,469 (32.2)	1,874 (30.7)		3,761 (30.6)	
Retiree/unemployed	2,436 (14.4)	1,202 (19.7)		1,694 (13.8)	
Lifestyle and comorbidities					
Number of family members N (%): living alone	1,999 (11.8)	1,790 (20.6)	0.13	1,894 (13.9)	0.07
2	5,792 (34.1)	2,939 (33.9)		3,817 (28.0)	

© 2021 Sarkar C et al. *JAMA Network Open.*

3	4,167 (24.6)	2,001 (23.1)		3,528 (25.9)	
≥4	5,010 (29.5)	1,941 (22.4)		4,398 (32.3)	
Smoking status N (%): Nonsmoker/past smoker	14,756 (87.0)	7,482 (86.5)	0.006	11,355 (83.4)	0.05
Current smoker	2,212 (13.0)	1,165 (13.5)		2,265 (16.6)	
Alcohol consumption N (%): Never/former	12,757 (75.2)	6,871 (80.7)	0.07	10,132 (74.8)	0.03
1-3/mo	3,060 (18.0)	1,053 (12.4)		2,301 (17.0)	
1-3/wk to daily	1,151 (6.8)	588 (6.9)		1,109 (8.2)	
Religion N (%): None	11,965 (70.5)	6,069 (70.0)	0.05	10,076 (73.9)	0.05
Christianity/Roman Catholicism	2,973 (17.5)	1,286 (14.8)		1,880 (13.8)	
Buddhism/Others	2,030 (12.0)	1,311 (15.1)		1,678 (12.3)	
Self-reported coronary heart disease N (%): No	16,591 (97.8)	8,099 (95.5)	0.06	13,197 (97.4)	0.01
Yes	377 (2.2)	385 (4.5)		349 (2.6)	
Self-reported High cholesterol level N (%): No	15,718 (92.6)	7,597 (89.6)	0.05	12,691 (93.7)	0.02
Yes	1,250 (7.4)	887 (10.5)		855 (6.3)	
Other neighbourhood environment					
Residential building age N (%): year built (1945-80)	3,638 (21.4)	2,176 (25.1)	0.06	3,102 (22.8)	0.03
1981-95	7,107 (41.9)	3,758 (43.4)		5,935 (43.6)	
Post-1995	6,223 (36.7)	2,728 (31.5)		4,588 (33.7)	
Public transport density within 402 m (units/Km^2) (Median, interquartile range)	37.8 (32.9)	38.0 (33.2)	0.04	38.1 (34.8)	0.03
Public transport density within 805 m (units/Km^2) (Median, interquartile range)	36.8 (22.4)	37.6 (22.9)	0.07	37.2 (24.3)	0.04
Terrain within 402 m (Mean slope in degrees) (Median, interquartile range)	5.5 (9.5)	5.8 (9.5)	0.02	5.3 (9.1)	<0.001
Terrain within 805 m (Mean slope in degrees) (Median, interquartile range)	7.4 (8.9)	7.5 (9.2)	0.03	7.0 (8.7)	<0.001
Neighbourhood cohesion N (%): Low	1,273 (7.5)	536 (6.2)	0.03	1,071 (7.9)	0.02
Medium	12,827 (75.6)	6,521 (75.2)		10,415 (76.4)	
High	2,868 (16.9)	1,611 (18.6)		2,147 (15.8)	
Proportion of working population in Neighbourhood (Mean, SD)	49.4 (6.3)	48.7 (6.2)	<0.001	49.4 (6.3)	0.006

© 2021 Sarkar C et al. *JAMA Network Open.*

Housing environment variables					
Floor area, m^2 (Mean, SD)	43.2 (14.4)	40.9 (15.2)	<0.001	42.9 (15.0)	<0.001
Housing units per building block (Mean, SD)	467.2 (296.7)	491.2 (315.0)	0.08	460.9 (300.2)	<0.001
Residential unit density within 402 m (units/Km^2) (Mean, SD)	38,025.9 (14,570.1)	38,465.4 (14,515.8)	0.03	37,073.7 (14,561.5)	<0.001
Residential unit density within 805 m (units/Km^2) (Mean, SD)	29,102.9 (11,325.1)	29,055.1 (11,139.8)	<0.001	28,398.0 (11,173.5)	<0.001
Floor level N (%): 0-5	3,156 (18.6)	1,600 (18.5)	0.04	2,589 (19.0)	0.02
6-10	3,207 (18.9)	1,828 (21.1)		2,758 (20.2)	
11-20	5,100 (30.1)	2,763 (31.9)		4,066 (29.8)	
21+	5,505 (32.4)	2,480 (28.6)		4,224 (31.0)	

[a]Cohen's *w* effect size for categorical variable: 0.1, small; 0.3, medium; 0.5, large.
[b]Cohen's *d* effect size for continuous variable: 0.2, small; 0.5, medium; 0.8, large.
[c]Comparison of the baseline characteristics of the analytic and excluded sample due to missingness on covariates.
[d]Comparison of the baseline characteristics of the analytic and excluded sample due to loss to follow-up.
[e]HK $1.0 to US $0.13.

© 2021 Sarkar C et al. *JAMA Network Open.*

eTable 2. Full Multilevel models of longitudinal association between multiple housing environment exposures and depressive sequelae among FAMILY Cohort participants aged 16 years or older across two waves (N = 16 968).

	**Probable major depression**		**Depressive Symptoms**	
	OR (95% CI)	p-value	OR (95% CI)	p-value
Residential density				
Floor area, m^2 (per IQR)	0.84 (0.71,0.98)	0.03	0.93 (0.86,1.00)	0.04
Housing units per building block (per IQR)	1.13 (0.91,1.40)	0.28	1.07 (0.97,1.19)	0.17
Neighbourhood residential density, units/Km^2 (402 m, per IQR)	0.94 (0.82,1.09)	0.45	1.00 (0.94,1.07)	0.98
Socio-demographic covariates				
Age in years	0.98 (0.97,0.99)	0.003	0.98 (0.98,0.99)	<0.001
Sex (Ref: Male)				
Female	2.66 (2.01,3.50)	<0.001	1.80 (1.61,2.01)	<0.001
Marital status (Ref: Never married)				
Married	0.68 (0.48,0.97)	0.03	0.92 (0.79,1.07)	0.29
Widowed/Divorced /separated	1.12 (0.70,1.81)	0.63	1.53 (1.23,1.91)	<0.001
Personal income in HK$ (Ref: ≤5 000)[a]				
5 000-9 999	0.77 (0.55,1.09)	0.14	0.96 (0.82,1.12)	0.59
10 000-14 999	0.72 (0.48,1.07)	0.10	0.89 (0.75,1.06)	0.18
≥15 000	0.66 (0.44,0.98)	0.04	0.78 (0.66,0.94)	0.008
Educational qualification (Ref: Primary)				
Secondary	0.90 (0.66,1.23)	0.50	0.84 (0.73,0.97)	0.02
Tertiary or above	0.65 (0.43,1.00)	0.05	0.85 (0.71,1.02)	0.08
Employment status (Ref: Employed)				
Homemaker/student/others	0.81 (0.59,1.12)	0.21	0.90 (0.78,1.03)	0.13
Retiree/unemployed	1.51 (1.00,2.26)	0.05	1.18 (0.97,1.43)	0.10
Lifestyle and comorbidities				
Number in family, Ref: Living alone				
2	0.85 (0.60,1.20)	0.37	0.84 (0.72,0.99)	0.04
3	0.78 (0.54,1.14)	0.20	0.82 (0.69,0.97)	0.02
≥4	0.93 (0.65,1.35)	0.71	0.94 (0.79,1.12)	0.51
Smoking status (Ref: Non-smoker/past smoker)				
Smoker	1.76 (1.25,2.47)	0.001	1.26 (1.08,1.46)	0.003
Alcohol intake frequency (Ref: Never/former drinker)				
Occasional up to 1-3 per month	2.08 (1.59,2.72)	<0.001	1.70 (1.51,1.92)	<0.001
1-3 per week up to daily	1.57 (0.97,2.56)	0.07	1.48 (1.21,1.80)	<0.001
Religion, Ref: None				

© 2021 Sarkar C et al. *JAMA Network Open.*

Christianity/Roman Catholicism	1.54 (1.16,2.03)	0.003	1.31 (1.15,1.48)	<0.001
Buddhism/Others	1.51 (1.11,2.06)	0.01	1.36 (1.18,1.57)	<0.001
Coronary heart disease (Yes vs. No)	2.53 (1.44,4.43)	0.001	1.89 (1.41,2.54)	<0.001
High cholesterol (Yes vs. No)	1.63 (1.09,2.43)	0.02	1.67 (1.40,1.99)	<0.001
Residential environment				
Residential building age, Ref: Old built (1945-80)				
Medium built (1981-95)	0.92 (0.67,1.27)	0.62	0.99 (0.85,1.16)	0.92
New built (post-1996)	1.00 (0.71,1.41)	0.99	1.11 (0.94,1.30)	0.21
Floor level (Ref: level 0-5)				
level 6-10	0.73 (0.50,1.05)	0.09	1.12 (0.94,1.32)	0.20
level 11-20	0.96 (0.70,1.32)	0.81	0.99 (0.85,1.16)	0.94
> level 20	0.84 (0.60,1.16)	0.29	0.99 (0.84,1.16)	0.88
Density of public transit (402 m),Ref: Q1				
Q2	1.09 (0.80,1.49)	0.59	0.97 (0.84,1.12)	0.65
Q3	1.13 (0.83,1.55)	0.44	0.87 (0.75,1.01)	0.07
Q4	1.02 (0.73,1.44)	0.89	1.06 (0.91,1.23)	0.47
Terrain (Mean slope)	1.01 (0.99,1.02)	0.53	1.01 (1.00,1.01)	0.13
Neighbourhood cohesion, Ref: Low				
Medium	0.30 (0.22,0.41)	<0.001	0.39 (0.33,0.46)	<0.001
High	0.15 (0.10,0.23)	<0.001	0.26 (0.22,0.32)	<0.001
Proportion of working population in Neighbourhood, Ref: Q1				
Q2	1.06 (0.79,1.44)	0.69	1.04 (0.89,1.20)	0.64
Q3	1.04 (0.73,1.47)	0.84	1.01 (0.86,1.19)	0.93
Q4	1.14 (0.80,1.62)	0.47	1.09 (0.92,1.28)	0.32

[a]HK $1.0 to US $0.13.

© 2021 Sarkar C et al. *JAMA Network Open.*

eTable 3. Full Multilevel models of longitudinal association between composite housing environment exposures and depressive sequelae using multiple imputation to impute loss to follow-up and missingness across key covariates (N = 39 276).

Composite housing environment	Probable major depression		Depressive symptoms	
	OR (95% CI)	p-value	OR (95% CI)	p-value
**Model 1**[a]				
Floor area, m^2 (per IQR)	0.83 (0.74,0.92)	<0.001	0.91 (0.86,0.95)	<0.001
Housing units per building block (per IQR)	1.15 (1.02,1.30)	0.03	1.10 (1.03,1.17)	0.003
Neighbourhood residential density, units/Km^2 (402 m, per IQR)	0.91 (0.83,1.00)	0.05	0.98 (0.93,1.02)	0.30
**Model 2**[b]				
Floor area, m^2 (per IQR)	0.91 (0.82,1.01)	0.07	0.95 (0.90,0.99)	0.03
Housing units per building block (per IQR)	1.08 (0.95,1.23)	0.24	1.07 (1.01,1.14)	0.03
Neighbourhood residential density, units/Km^2 (402 m, per IQR)	0.92 (0.84,1.01)	0.07	0.98 (0.94,1.03)	0.44
**Model 3**[c]				
Floor area, m^2 (per IQR)	0.90 (0.81,1.00)	0.049	0.93 (0.89,0.98)	0.01
Housing units per building block (per IQR)	1.07 (0.93,1.23)	0.32	1.07 (1.00,1.15)	0.06
Neighbourhood residential density, units/Km^2 (402 m, per IQR)	0.89 (0.81,0.98)	0.01	0.96 (0.92,1.01)	0.13

[a]Model 1 adjusted for age and sex.
[b]In addition to age and sex, model 2 was adjusted for other socio-demographic characteristics (marital status, employment status, educational level, income), lifestyle (smoking status, alcohol intake frequency, number of family members and current religion) and comorbidities (cardiac heart disease, high cholesterol level).

© 2021 Sarkar C et al. *JAMA Network Open.*

[c]Fully adjusted model 3 comprised the factors in models 1 and 2 plus the residential environment (residential building age, floor level, density of public transport, terrain, neighborhood cohesion and proportion of working population in the neighborhood).
The residential environment (neighbourhood residential density, density of public transport and terrain) was measured within 402 m of street catchment of participants' geocoded residences.

© 2021 Sarkar C et al. *JAMA Network Open.*

eTable 4. Multilevel negative binomial regression models of longitudinal association between housing environment exposures and PHQ-9 score among FAMILY Cohort participants aged 16 years or older across two waves (N = 16 968).

	Single housing environment models*		Composite model with multiple housing environment**	
	IRR (95% CI)	p-value	IRR (95% CI)	p-value
**Model 1[a]**				
Floor area, m^2 (per IQR)	0.96 (0.93,0.98)	<0.001	0.97 (0.94,1.00)	0.03
Housing units per building block (per IQR)	1.08 (1.04,1.12)	<0.001	1.06 (1.02,1.11)	0.002
Neighbourhood residential density, units/Km^2 (402 m, per IQR)	1.01 (0.99,1.04)	0.40	1.00 (0.97,1.02)	0.84
**Model 2[b]**				
Floor area, m^2 (per IQR)	0.96 (0.93,0.99)	0.005	0.97 (0.94,1.00)	0.05
Housing units per building block (per IQR)	1.07 (1.03,1.12)	<0.001	1.06 (1.01,1.10)	0.01
Neighbourhood residential density, units/Km^2 (402 m, per IQR)	1.01 (0.98,1.04)	0.48	1.00 (0.97,1.03)	0.97

*****The housing environment variables (floor area, housing units per building block and neighbourhood residential density) were entered into the model separately in the single housing environment models.

******The housing environment variables were simultaneously entered into the model in the composite housing environment model.

[a]Model 1 adjusted for age and sex.

[b]Model 2 represents fully-adjusted models accounting for socio-demographics (age, sex, marital status, employment status, educational level, income), lifestyle (smoking status, alcohol intake frequency, number of family members and current religion), comorbidities (cardiac heart disease, high cholesterol level) and residential environment (residential building age, floor level, density of public transport, terrain, neighbourhood cohesion and proportion of working population in the neighbourhood).

© 2021 Sarkar C et al. *JAMA Network Open.*

The residential environment (neighbourhood residential density, density of public transport and terrain) was measured within 402 m of street catchment of participants' geocoded residences.

© 2021 Sarkar C et al. *JAMA Network Open.*

eTable 5. Multilevel models of longitudinal association between composite housing environment exposures and depressive sequelae among FAMILY Cohort participants aged 16 or older across two waves with residential environment measured within 0.50-mile (805 m) street catchment (N = 16 968).

**Composite housing environment**	Probable major depression		Depressive symptoms	
	OR (95% CI)	p-value	OR (95% CI)	p-value
**Model 1[a]**				
Floor area, m^2 (per IQR)	0.79 (0.68,0.92)	0.003	0.91 (0.84,0.97)	0.005
Housing units per building block (per IQR)	1.16 (0.97,1.39)	0.10	1.10 (1.00,1.20)	0.04
Neighbourhood residential density, units/Km^2 (805 m, per IQR)	0.99 (0.86,1.14)	0.86	0.95 (0.89,1.01)	0.09
**Model 2[b]**				
Floor area, m^2 (per IQR)	0.86 (0.74,0.99)	0.04	0.93 (0.87,1.00)	0.05
Housing units per building block (per IQR)	1.11 (0.92,1.33)	0.29	1.06 (0.97,1.17)	0.18
Neighbourhood residential density, units/Km^2 (805 m, per IQR)	0.99 (0.86,1.14)	0.91	0.95 (0.89,1.01)	0.08
**Model 3[c]**				
Floor area, m^2 (per IQR)	0.82 (0.70,0.97)	0.02	0.92 (0.85,0.99)	0.02
Housing units per building block (per IQR)	1.04 (0.84,1.30)	0.70	1.04 (0.94, 1.15)	0.47
Neighbourhood residential density, units/Km^2 (805 m, per IQR)	1.03 (0.88,1.19)	0.72	0.96 (0.90,1.02)	0.20

[a]Model 1 adjusted for age and sex.
[b]In addition to age and sex, model 2 was adjusted for other socio-demographic characteristics (marital status, employment status, educational level, income), lifestyle (smoking status, alcohol intake frequency, number of family members and current religion) and comorbidities (cardiac heart disease, high cholesterol level).

© 2021 Sarkar C et al. *JAMA Network Open.*

[c]Fully adjusted model 3 comprised the factors in models 1 and 2 plus the residential environment (residential building age, floor level, density of public transport, terrain, neighborhood cohesion and proportion of working population in the neighborhood).
The residential environment (neighbourhood residential density, density of public transport and terrain) was measured within 805 m of street catchment of participants' geocoded residences.

© 2021 Sarkar C et al. *JAMA Network Open.*

eTable 6. Results of the multinomial logit regression models of association of housing environment exposures and depressive symptoms at wave 2 by depressive symptoms at baseline (N = 16 968).

Depressive symptoms[a]	Single housing environment models*		Composite model with multiple housing environment**	
	RR (95% CI)	p-value	RR (95% CI)	p-value
Ref: No depressive symptoms at wave 2 and baseline				
Depressive symptoms at wave 2 but not at baseline				
Floor area, m^2 (per IQR)	0.88 (0.79,0.97)	0.009	0.89 (0.81,0.99)	0.03
Housing units per building block (per IQR)	1.16 (1.02,1.32)	0.02	1.12 (0.98,1.28)	0.10
Neighbourhood residential density, units/Km^2 (402 m, per IQR)	1.00 (0.91,1.09)	0.95	0.97 (0.89,1.07)	0.56
Depressive symptoms at wave 2 and baseline				
Floor area, m^2 (per IQR)	0.97 (0.86,1.10)	0.66	0.99 (0.87,1.13)	0.91
Housing units per building block (per IQR)	1.10 (0.92,1.32)	0.31	1.08 (0.90,1.31)	0.40
Neighbourhood residential density, units/Km^2 (402 m, per IQR)	1.05 (0.93,1.19)	0.41	1.04 (0.92,1.18)	0.51

*****The housing environment variables (floor area, housing units per building block and neighbourhood residential density) were entered into the model separately in the single housing environment models.
******The housing environment variables were simultaneously entered into the model in the composite housing environment model.
[a]Fully-adjusted models accounting for socio-demographics (age, sex, marital status, employment status, educational level, income), lifestyle (smoking status, alcohol intake frequency, number of family members and current religion), comorbidities (cardiac heart disease, high cholesterol level) and residential environment (residential building age, floor level, density of public transport, terrain, neighbourhood cohesion and proportion of working population in the neighbourhood). The residential environment (neighbourhood residential density, density of public transport and terrain) were measured within 402 m of street catchment of geocoded participants' residence.

© 2021 Sarkar C et al. *JAMA Network Open.*

eTable 7. Association of livable floor area with prevalent probable major depression and depressive symptoms by population sub-groups of sex, age and employment status at baseline (N = 16 968).

	n	Probable major depression[a]		Depressive symptoms[a]	
		OR (95% CI)	p-value	OR (95% CI)	p-value
**Sex**					
Male	7,640	0.88 (0.67,1.16)	0.36	0.90 (0.81,1.01)	0.07
Female	9,328	0.87 (0.70,1.07)	0.18	0.95 (0.86,1.05)	0.29
p-interaction			0.87		0.91
**Age groups**					
≤40	6,450	0.83 (0.61,1.13)	0.24	0.98 (0.86,1.11)	0.77
>40-≤60	7,201	0.70 (0.55,0.90)	0.01	0.87 (0.78,0.98)	0.02
>60	3,317	1.24 (0.96,1.61)	0.10	0.94 (0.80,1.09)	0.40
p-interaction			0.44		0.54
**Employment status**					
Employed	9,063	0.84 (0.65,1.07)	0.16	0.87 (0.78,0.96)	0.01
Unemployed, home maker, others	5,469	0.86 (0.64,1.15)	0.30	1.02 (0.90,1.16)	0.72
Retiree/Unemployed	2,436	1.00 (0.66,1.53)	0.99	0.97 (0.83,1.14)	0.73
p-interaction			0.71		0.02

[a]Fully-adjusted models accounting for socio-demographics (age, sex, marital status, employment status, educational level, income), lifestyle (smoking status, alcohol intake frequency, number of family members and current religion), comorbidities (cardiac heart disease, high cholesterol level) and residential environment (residential building age, floor level, density of public transport, terrain, neighbourhood cohesion and proportion of working population in the neighbourhood). The residential environment (neighbourhood residential density, density of public transport and terrain) were measured within 402m of street catchment of geocoded participants' residence.

© 2021 Sarkar C et al. *JAMA Network Open.*

eTable 8. Multilevel association of housing environment exposures with depressive sequelae among FAMILY Cohort participants aged 16 years or older who did not change residential address over the study period (N = 16 407).

	**Probable major depression**[a]		**Depressive symptoms**[a]	
	OR (95% CI)	p-value	OR (95% CI)	p-value
Housing environment				
Floor area, m^2 (per IQR)	0.82 (0.70,0.98)	0.03	0.91 (0.84,0.98)	0.02
Housing units per building block (per IQR)	1.12 (0.90,1.39)	0.31	1.05 (0.95,1.17)	0.33
Neighbourhood residential density, units/Km^2 (402 m, per IQR)	0.96 (0.83,1.11)	0.60	0.99 (0.93,1.06)	0.83

[a]Fully-adjusted models accounting for socio-demographics (age, sex, marital status, employment status, educational level, income), lifestyle (smoking status, alcohol intake frequency, number of family members and current religion), comorbidities (cardiac heart disease, high cholesterol level) and residential environment (residential building age, floor level, density of public transport, terrain, neighbourhood cohesion and proportion of working population in the neighbourhood). The residential environment (neighbourhood residential density, density of public transport and terrain) were measured within 402 m of street catchment of geocoded participants' residence. The housing environment variables were simultaneously entered into the model in the composite housing environment model.

© 2021 Sarkar C et al. *JAMA Network Open.*

eFigure. An illustration showing the attributes of housing exposures of multi-scalar residential space and density in the developed HKHED database; liveable floor area at household level, building units per block at building (meso-) level, and neighbourhood residential density within 402 m (0.25-mile) & 805 m (0.5-mile) residential catchments at macro-level.

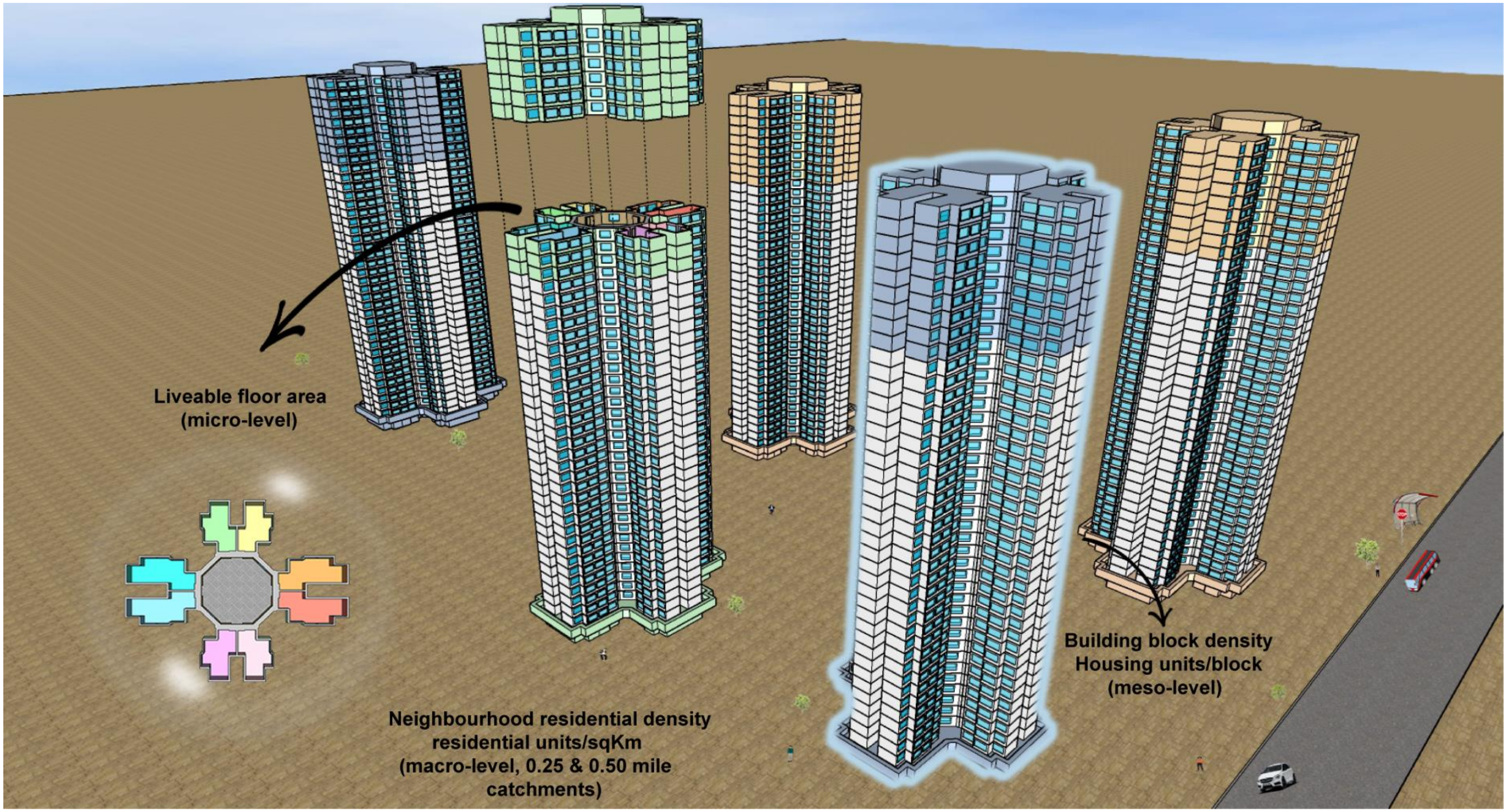

© 2021 Sarkar C et al. *JAMA Network Open.*
